# Poly(Ethylene Glycol) as a Scaffold for High-Affinity Open-Channel Blockers of the Mouse Nicotinic Acetylcholine Receptor

**DOI:** 10.1371/journal.pone.0112088

**Published:** 2014-11-11

**Authors:** Wan-Chen Lin, Stuart Licht

**Affiliations:** Department of Chemistry, Massachusetts Institute of Technology, Cambridge, Massachusetts, United States of America; Universidad de Castilla-La Mancha, Spain

## Abstract

High-affinity blockers for an ion channel often have complex molecular structures that are synthetically challenging and/or laborious. Here we show that high-affinity blockers for the mouse nicotinic acetylcholine receptor (AChR) can be prepared from a structurally simple material, poly(ethylene glycol) (PEG). The PEG-based blockers (**PQ1**–**5**), comprised of a flexible octa(ethylene glycol) scaffold and two terminal quaternary ammonium groups, exert low- to sub-micromolar affinities for the open AChR pore (measured via single-channel analysis of AChRs expressed in human embryonic kidney cells). **PQ1**–**5** are comparable in pore-binding affinity to the strongest AChR open-channel blockers previously reported, which have complex molecular structures. These results suggest a general approach for designing potent open-channel blockers from a structurally flexible polymer. This design strategy involves simple synthetic procedures and does not require detailed information about the structure of an ion-channel pore.

## Introduction

Ion channels play important roles in diverse physiological processes, including neuronal signaling [1], cardiac rhythm setting [2], and insulin secretion [3]. Small molecules that positively or negatively modulate ion channel activities are commonly used as drugs or research tools. Open-channel blockers, a class of ion channel inhibitors that sterically occlude the open pore of an active channel, have been used in both clinical treatment and basic research. For instance, slow-channel congenital myasthenic syndromes, a type of neuromuscular junction dysfunctions caused by abnormally prolonged opening of the nicotinic acetylcholine receptor (AChR), are treated by long-lived blockers for the AChR [4]–[6]. Memantine, an open-channel blocker for the *N*-methyl-D-aspartate receptor, is used for the treatment of Alzheimer's disease [7]. Due to the versatility and clinical potential of open-channel blockers, it would be desirable to develop new strategies for more convenient and straightforward design of these agents.

Rational design of open-channel blockers has been challenging due to the limited availability of high-resolution structures for the ion-conducting pores. Open-channel blockers are believed to bind within the transmembrane pore via noncovalent interactions with the hydrophobic pore-lining residues. Drugs that exhibit appreciable blockade affinities mostly have rigid and/or complex structures, suggesting that these molecules have well-defined binding geometries within their target sites. Without a detailed structure of the open channel pore, blocker design or optimization cannot be carried out via the typical structure-based approach, in which a ligand needs to be docked in the binding site. Even when a high-resolution structure of the target channel is available, it remains difficult to predict how variations in the chemical structure would affect the kinetics of blocker association and dissociation.

**Figure 1:**
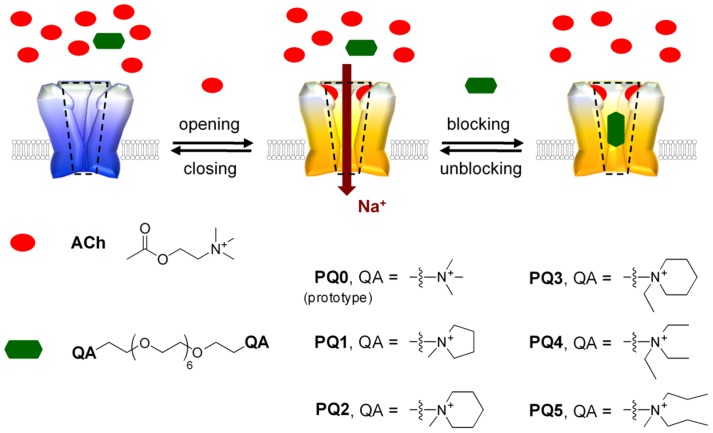
Structure and mode of action of PEG-based open-channel blockers (PQ1–PQ5) for the AChR.

Here we report the synthesis and functional characterizations of polyethylene glycol (PEG)-based open-channel blockers of the nicotinic AChR that both achieve high pore-binding affinities (comparable to those of natural pore-blocking toxins) and possess simple, readily accessible molecular structures. These PEG-based blockers (**PQ1**–**PQ5**; Figure 1) are distinct from conventional AChR blockers in that they do not have a rigid, complex core scaffold. This flexibility in molecular shape may facilitate the development of high-affinity blockers when a high-resolution structure for the open channel pore is not available, providing new opportunities for future blocker discoveries.

## Materials and Methods

### Synthesis of blockers PQ1–5 (general procedure)

The diiodide derivative of octa(ethylene glycol) [8] (120–140 mg, 1 equiv.) was dissolved in anhydrous CH_3_CN (1.5 mL) and was added the reacting tertiary amine (1.5 mL). The reaction mixture was stirred at 25°C (for **PQ1**) or 75°C (for **PQ2**–**PQ5**) for 16–24 h. The solvent and the reactant amine were subsequently removed by rotary evaporation. The residue was purified by a short silica gel column to provide the final product. Synthetic protocols and product characterizations are included in File S1.

### Cell culture and transfection

The cDNA clones of adult mouse muscle AChR subunits (α, β, δ, ε) in the vector pRBG4 were generously provided by Professor Anthony Auerbach (University at Buffalo, NY) [9]. The α subunit contains the previously described V433A background mutation which does not affect gating kinetics and is referred to as wild-type [9]. Human embryonic kidney cells (HEK-293 cells, ATCC CRL-1573) were transiently transfected by calcium phosphate precipitation [9]. In brief, HEK-293 cells were maintained in Dulbecco's Minimum Essential Media (Invitrogen) supplemented with 10% Fetal Bovine Serum (Invitrogen) at 37°C and 5% CO_2_. Cells were plated 24 h before transfection to reach a confluence of 40–60%. A total of 3.5 µg DNA per 35-mm culture dish (α:β:δ:ε = 2∶1∶1∶1) was used. Transfecting HEK-293 cells with this ratio of subunit DNAs has previously been demonstrated to enable the expression of muscle-type AChRs exhibiting the characteristic gating kinetics of the adult isoform [9], [10]. The medium was changed 24 h after the addition of DNA, and patch-clamp recordings were carried out 20–36 h thereafter.

### Recording and analysis of single-channel events

Single-channel currents from AChRs were recorded using patch-clamp techniques in the cell-attached configuration. For dose-dependence measurements, a holding potential of +70 mV was applied. Kinetic analysis of single-channel currents was carried out using the QuB suite [11], [12]. The currents were idealized using the segmented k-means (SKM) hidden Markov algorithm at full bandwidth (10 kHz) [13]. Kinetic modeling of the idealized intervals was performed using the maximum interval likelihood (MIL) method [11], [12]. Additional details of recording and data analysis are provided in File S1.

**Figure 2:**
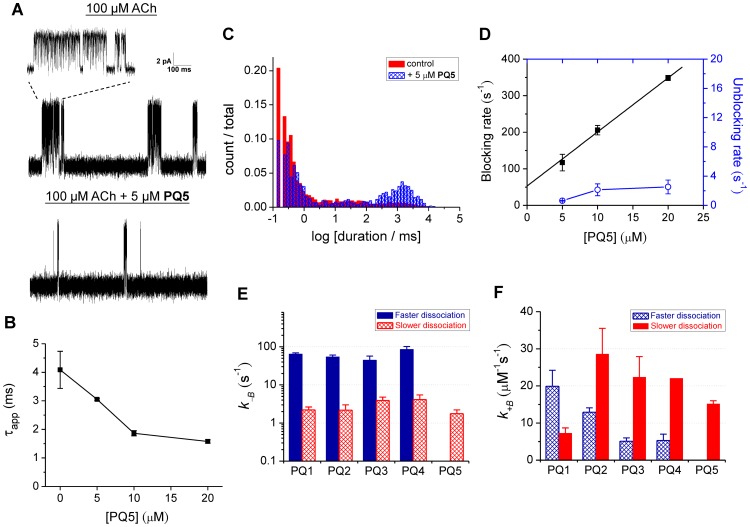
Kinetic characterizations of AChR blockade by PQ1–5. (A) Single-channel currents of the AChR in the presence of 100 µM Ach ±5 µM **PQ5**. Recordings were carried out in the cell-attached configuration held at +70 mV. Currents are displayed as upward deflections. (B) Dose-dependent decrease in the apparent mean open time (τ_app_) of the AChR. Data are plotted as mean ± SEM (n = 3–4). (C) Closed-dwell histograms (duration in ms) at 100 µM ACh in the presence (blue) and absence (red) of 5 µM **PQ5**. (D) Blocking (filled symbols) and unblocking (open symbols) rates of **PQ5** estimated by MIL analysis [10], [11]. Data are plotted as mean ± SEM (n = 3). The blocking rate constant (*k_+B_*) of **PQ5** can be estimated from the slope of the least-squares linear fit for the blocking rates (black line). (E) The unblocking rate constants (*k_–B_*; mean + SEM, n = 6–8) of **PQ1**–**5** for fast (blue) and slow (red) dissociation modes estimated from MIL analysis. (F) The blocking rate constants of **PQ1**–**5** for fast (blue) and slow (red) dissociation modes estimated from MIL analysis (as illustrated in panel D). Error bars represent the standard errors of the linear fits. The results of MIL analysis (τ_app_s, blocking rates, and unblocking rates at different doses) for **PQ1**–**4** are summarized in Figure S3.

MIL analysis on 2–4 cells was performed to determine the apparent mean open time (τ_app_), blocking rate, and unblocking rate for each measuring condition (reported as mean ± SEM). Uncertainties in the blocking rate constant (*k_+B_* values in Figure 2F) are reported as the standard errors in the least squares fits.

## Results and Discussion

To address the technical challenges of structure-based blocker design, we have previously proposed a new strategy, using the muscle-type AChR as the test system, to minimize the need for complex molecular scaffolds and detailed structure-function predictions [8]. The proposed AChR blockers comprise: (1) a flexible polymeric backbone that is capable of adopting favorable conformations to interact with the pore interior; and (2) one or more quaternary ammonium (QA) groups that interact with the transmembrane electric field and direct the molecule to target the open pore [8]. Poly(ethylene glycol) (PEG) was chosen as the polymeric backbone because of its structural flexibility and simplicity. It is a widely used linker component in a variety of bioconjugates, including tethered agonists or blockers for ion channels [14]–[16].

The proposed strategy was first tested using a series of PEG-trimethylammonium conjugates [8]. In the initial proof-of-concept study, the prototype PEG-based blockers (containing 4–13 ethylene oxide units in the backbone) exhibited open-channel blockade kinetics that varied with the length of the polymer backbone, suggesting that the PEG moiety itself exerts energetically favorable interactions with the channel pore. However, these prototype blockers bound to the AChR pore with only moderate affinities (K_d_ = 8–850 µM), which are insufficient for applications that require high-affinity blockade. To further enhance pore-binding affinity of a PEG-based blocker, we attempted to identify other structural components that could interact effectively with the open AChR pore when appended to the polymer.

Several lines of evidence suggest that the binding affinity of a PEG-based blocker could be enhanced by tuning the QA structure. Previous studies on symmetric tetraalkylammonium salts [17], lidocaine derivatives [18], and *p*-phenylene-polymethylene bis-ammonium compounds [19] showed that replacing the trimethylammonium moiety of the parent blocker with a bulkier QA enhanced the blocker's potency. These studies suggest the presence of a QA-binding pocket which can accommodate QA groups bulkier than trimethylammonium. Moreover, these results imply that in addition to the core blocker scaffold, the substituents of QA are also involved in interacting with the hydrophobic environment of the AChR pore. If the trimethylammonium groups of our prototype blockers target the same QA-binding pocket, enhancement of blocker affinities could be achieved by modifying the QA groups.

To test this hypothesis, we characterized the blockade kinetics of a series of PEG-QA conjugates (**PQ1**–**PQ5**; Figure 1) using patch-clamp electrophysiology. These conjugates were prepared from a short PEG, octa (ethylene glycol), through a three-step conversion (File S1). The chosen QA groups are small relative to the backbone length, and the hydrodynamic radii of these compounds are therefore expected to be similar. The kinetics and microscopic equilibrium affinity of channel blockade were determined by analyzing the acetylcholine (ACh)-elicited currents at the single-channel level. Channel activities were measured at 100 µM ACh in the absence or presence of 5, 10, or 20 µM of PEG-QA. At 100 µM ACh, the single-channel currents are displayed as clusters of opening events separated by long desensitized dwells in which the ACh-bound channel adopts a non-conducting conformation (Figures 2A and S1). The brief gaps within the clusters represent channel closing events.

Typical open-channel blockers follow a sequential blockade mechanism: the channel opens in response to ACh binding, and the blocker reversibly binds and dissociates from the transmembrane pore before the channel closes (Figure 1 and File S1). With that mechanism, a dose-dependent decrease in the apparent mean open time (τ_app_, the mean duration of the identified opening events) is observed. A new population of non-conducting blockade events, in addition to the brief closing and the long desensitized dwells, is also observed. These blockade events manifest themselves as a new component in the closed-time histogram. The relative area of the blockade component increases with increasing blocker concentration (reflecting the concentration-dependent rate of pore blockade), while the lifetime of this component (τ_B_) is independent of blocker concentration (reflecting the unimolecular nature of blocker dissociation). These kinetic features allow the blocking and unblocking rate constants (*k_+B_* and *k_–B_*, respectively) to be determined by fitting of the dwell times under each set of conditions (File S1).

As shown in Figure 2, **PQ5** exhibits the characteristics of sequential open-channel blockade, allowing the measurement of its blocking and unblocking rate constants. **PQ5**, which has an *N*-methyldipropylammonium group at each end of the PEG scaffold, causes a dose-dependent decrease in τ_app_ (Figure 2B). In the presence of 5 µM **PQ5**, a new component arises in the closed-time histogram (Figure 2C). The lifetime of this component does not change with the concentration of **PQ5**, but the relative area of this component increases dose-dependently. This kinetic component can therefore be interpreted as being associated with **PQ5**-mediated pore blockade. The blocking and unblocking rates under each condition were estimated by fitting the dwell times using the Maximum Interval Likelihood (MIL) algorithm [11], [12]. As shown in Figure 2D, the blocking rate increases linearly with the concentration of **PQ5**, whereas the unblocking rate remains approximately constant over the tested concentration range. The blocking rate constant was estimated from the slope of the linear fit for Figure 2D (*k_+B_* = 15±1 µM^−1^s^−1^), and the unblocking rate constant was calculated by averaging the unblocking rates at all tested concentrations (*k_–B_* = 2±1 s^−1^; n = 8).

The hypothesis that **PQ5** binds within the transmembrane pore is further supported by the voltage-dependent changes in τ_app_ and *k_+B_* (Figure S2). In general, blockade is sensitive to changes in membrane potential if the molecule is charged and the site of action is located in the transmembrane electric field. In the presence of **PQ5**, τ_app_ decreases with hyperpolarizing (i.e. more negative) membrane potential. The value of *k_+B_* also increases with membrane hyperpolarization, consistent with a mechanism in which the positively charged PEG-QA interacts with the transmembrane electric field.

The results of single-channel analysis for **PQ1**–**5** are summarized in Figures 2E and 2F (see Figure S3 for analysis details). Surprisingly, two closed-time components characteristic of open-channel blockade are found with **PQ1**–**4** (Figure S3), indicating that **PQ1**–**4** might exert two modes of blockade. In contrast, only one blocked component is identified for **PQ5** in the closed-time histogram. The two modes are designated by their dissociation rate constants: *k_–B_* is 45–85 s^−1^ for the faster-dissociation mode and 2–4 s^−1^ for the slower-dissociation mode (Figure 2E). The rate constants for entering these modes (i.e. the *k_+B_*s) vary with the QA structure in **PQ1**–**4**. The *k_+B_* of the faster-dissociation mode decreases with increasing size of the QA group (Figure 2F). The faster-dissociation mode is the predominant blockade mechanism for **PQ1**, which has a smaller QA group (*N*-methylpyrrolidium) at each end of PEG; the slower-dissociation mode is favored by **PQ2**–**5**. Measuring the blocking and unblocking rate constants for **PQ1**–**5** allows the equilibrium dissociation constants (K_d_ = *k_–B_*/*k_+B_*) to be determined. The K_d_ values of the predominant blockade mode are 3±1, 0.08±0.09, 0.17±0.12, 0.19±0.15, and 0.12±0.09 µM for **PQ1**–**5**, respectively. Due to limited variation in the QA structure over the series, the modest substituent-dependent changes observed in blockade kinetics may be insufficient for a quantitative structure-activity analysis. However, the affinities of **PQ1**–**5** are all higher than that of the prototype blocker (**PQ0** in Figure 1; K_d_ = 9±2 µM; ref. 8), consistent with our hypothesis that the QA substituents can interact with the channel pore.

**Figure 3:**
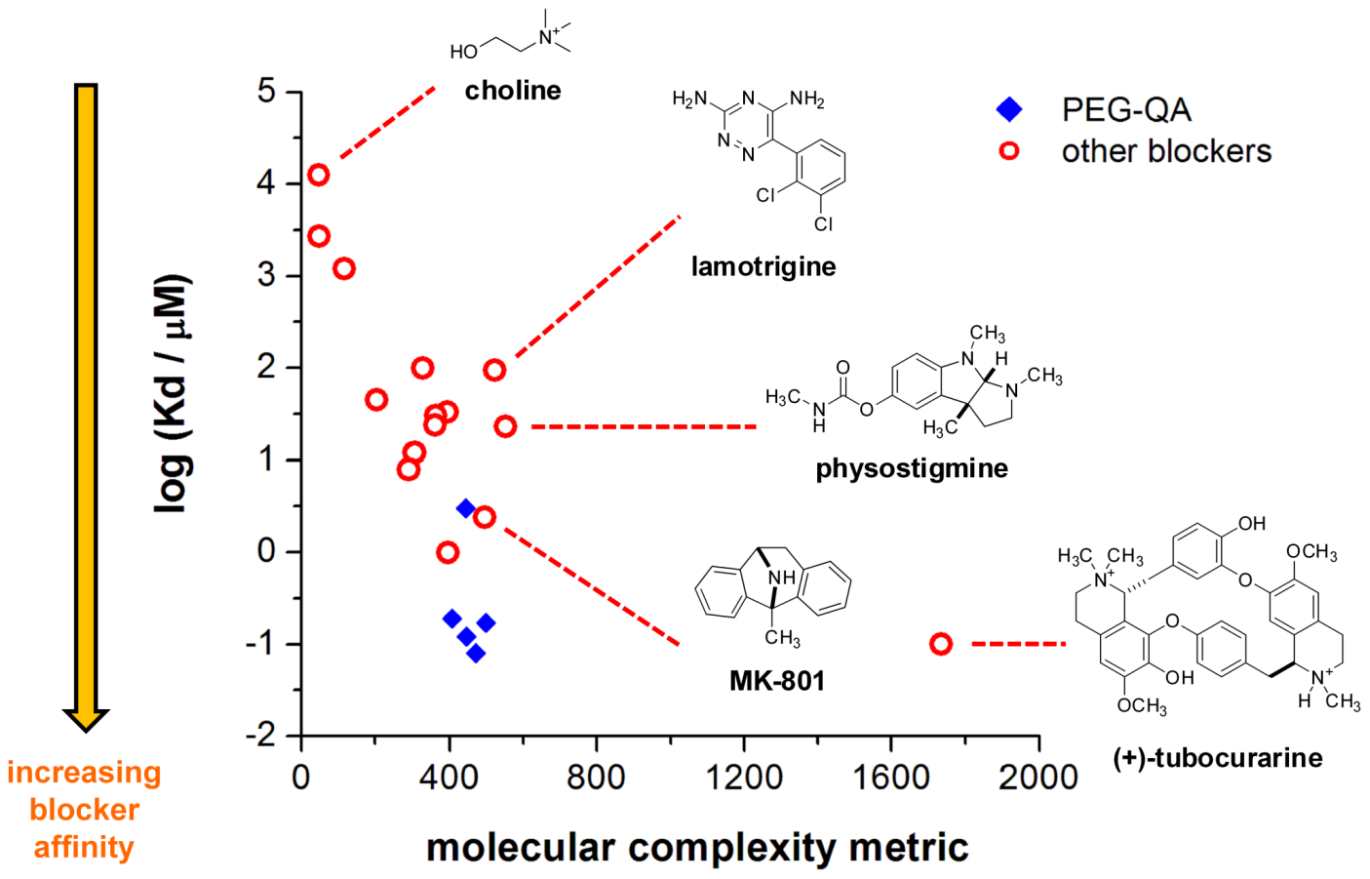
A comparison of non-PEG-based and PEG-based blockers with respect to affinity and molecular complexity. Affinity is expressed as log (K_d_). Non-PEG-based blockers are shown as red circles, while PEG-based blockers are shown as blue diamonds. The plotted data are obtained from Table S1 and references therein.

The pore-binding affinities of PEG-QAs are comparable to those of the most potent AChR blockers characterized previously. The dominant factor determining the high affinities of **PQ2**–**5** is their low *k_–B_*s (2–4 s^−1^). Although many ammonium compounds have been reported to block the open AChR pore, most of their K_d_s are within micromolar or millimolar ranges and their *k_–B_*s are relatively high (>100 s^−1^; Table S1). To our knowledge, very few blockers bind the open AChR pore with sub-micromolar affinities. Compounds that do exhibit similar blockade kinetics and/or affinities to **PQ1**–**5**, such as (+)-tubocurarine [20] and MK-801 [21], are structurally complex and synthetically non-trivial. Qualitatively, **PQ1**–**5** are structurally less complex than the previously reported high-affinity blockers (Figure 3 and Table S1). This comparison can be more quantitatively described using the Bertz index for molecular complexity, an unbiased comparator that correlates well with structural features associated with complex molecules, such as size, number of rings, branching and symmetry [22], [23]. Based on this index, **PQ2**–**5** have similar pore affinities to that of (+)-tubocurarine but are approximately 3.5-fold less complex in the molecular structure (Figure 3 and Table S1), suggesting that PEG is a more efficient scaffold than those of the conventional AChR blockers on a complexity basis. Consistent with this interpretation, **PQ2**–**5** are substantially more potent (>10-fold in affinity) than the previously characterized blockers that have comparable molecular complexity, including physostigmine [24], MK-801 [21], and lamotrigine [25] (Figure 3 and Table S1).

Various QA compounds, including the prototype PEG-QA (**PQ0**), have been previously reported as agonists for the muscle-type AChR [8], [19], [26]–[28]. **PQ1**–**5** are also capable of inducing AChR openings to some degree. The agonist potencies of PEG-QAs correlate roughly inversely with the size of their QA groups (except **PQ4**; Figure S4). Notably, **PQ3** and **PQ5** are poor agonists as judged by the low channel open probabilities and brief opening durations observed with these compounds alone (Figure S4). The low agonist potencies of **PQ3** and **PQ5** may be attributable to their bulky QA groups, which may cause reduced affinities for the agonist-binding pocket [17] and/or interfere with receptor conformational changes required for channel gating [29]. Open-channel blockers with low agonist potencies may be favorable in some applications, since their pharmacological effects are simpler and more predictable.

The present work demonstrates that, in addition to the polymer length, the QA structure of a PEG-based blocker is another key component for determining its pore-binding affinity. Replacing trimethylammonium groups with bulkier QAs in the prototype enhanced the blocker's affinity, supporting the hypothesis that a hydrophobic QA-binding pocket is present in the open AChR pore. Nicotinic AChRs are members of the pentameric Cys-loop receptor superfamily [29]. A structural investigation of QA-mediated open-channel block in *Gloeobacter* ligand-gated ion channel (GLIC), a prokaryotic homolog of vertebrate Cys-loop receptors, has been previously performed using X-ray crystallography [30]. The study revealed a QA-binding site located in a cavity between the hydrophobic and hydrophilic part of the pore, in which the QA fills the space with its alkyl substituents, thus occluding the open pore. This binding mode suggests that hydrophobic interactions play an important role in QA binding, consistent with the size-dependent enhancement of QA-mediated blockade. Due to the structural, functional, and pharmacological similarities between GLIC and the AChR [30], [31], the same QA-binding site may also be present in the AChR pore and be targeted by PEG-QAs.

Together with our previous study [8], the present work suggests that using PEG as the scaffold allows straightforward and convenient preparation of high-affinity AChR blockers. By simply linking two QA groups with an appropriately sized PEG, a new type of AChR blocker with low- to sub-micromolar affinities can be prepared. The simple preparation of these compounds will facilitate further expansion of the molecular diversity by varying the QA structure or the PEG size, providing a potential strategy for discovering even more potent and/or selective blockers for the AChR. Improved AChR blockers may find applications in neurobiology as pharmacological tools or as targeting groups of activity-based probes [32]. In addition, since the pore of other Cys-loop receptors also contain an extracellular vestibule lined by non-polar residues [33], properly functionalized PEGs are likely to serve as blockers for these ion channels as well. PEG, a simple and commonly used building block for bioconjugates, is therefore a promising scaffold for the future design of high-affinity channel blockers.

## Supporting Information

Figure S1 **Characterization of single-channel currents at 100 µM ACh (i.e. the control condition).** (A) A representative trace showing clusters of opening/closing events separated by desensitized dwells. Currents are shown as upward deflections. Pipette potential was held at +70 mV. (B) The kinetic model used for fitting the acquired single-channel events (by MIL). C, closed state; O, open state; D_1_, D_2_, D_3_, desensitized states (ranked in the order of increasing lifetime). (C) Representative histograms (duration in ms) for the closed-time (left) and the open-time (right) distributions. Each component in the closed-time histogram is designated by the corresponding state in the kinetic model. The solid curves represent the overall fit for the histogram, and the red dashed curves indicate the fits for individual components. Additional details about data acquisition and analysis can be found in File S1 (section 1.3).
(TIF)

Figure S2 **Voltage dependence of AChR blockade by PQ5 (20 µM).** (A) A representative plot of τ_app_ vs. membrane potential. (B) A representative plot of ln(*k_+B_*) vs. membrane potential. The data points in panel B are fitted by least-squares linear regression. Recordings were carried out in the cell-attached configuration held at +50, +70, +100, and +130 mV. The membrane potential (i.e. the x-axes in both panels) at each holding potential (V_hold_) was estimated as V_rest_ – V_hold_, in which V_rest_ (resting potential) was obtained from the linear plot of current amplitude vs. V_hold_ [8]. Note that the y-axes in both plots are truncated in order to highlight the data.
(TIF)

Figure S3 **Kinetic characterizations of AChR blockade mediated by PQ1 (A), PQ2 (B), PQ3 (C), and PQ4 (D).** Recordings were carried out in the cell-attached configuration held at +70 mV, with 100 µM ACh and various doses of PQ compounds in the recording pipette. The analyses of single-channel currents for **PQ1**–**4** were the same as those for **PQ5** (Figure 2 and related text). Each PQ compound caused a dose-dependent decrease in the apparent mean open time (τ_app_, left) and dose-dependent increases in the relative fractions (but not the duration lifetimes) of two closed-time components. These closed-time components may thus be interpreted as two modes of open-channel blockade. MIL analysis on dwell-time histograms provided the blocking rates (middle) and unblocking rates (right) for these two modes (blue: faster dissociation, i.e. shorter blockade durations; red: slower dissociation, i.e. longer blockade durations). Data are plotted as mean ± SEM (n = 2–4). The blocking rate constants (*k_+B_*s) were obtained from the slopes of least-squares linear fitting for the blocking rates, and the unblocking rate constants (*k_–B_*s) were calculated by averaging the unblocking rates at all tested concentrations. The resulting rate constants are summarized in Figures 2E and 2F. Due to the complexity in closed-time histograms, the data of slower dissociation mode at 20 µM **PQ4** were not included in this analysis.
(TIF)

Figure S4
**The agonist potencies of PQ1–5.** (A) Representative traces of single-channel recordings in the presence of individual PEG-QA (10 µM in the pipette solution without other agonists). Pipette potential was held at +70 mV, and the currents are shown as upward deflections. (B) The effect of QA structure on the open probability of the AChR (defined as 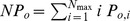, where *N*_max_ is the maximal number of simultaneously open channels and *P_o,i_* is the fraction of time in which *i* channels open simultaneously). Data are plotted in log-scale in order to present the low *NP_o_* values of **PQ3** and **PQ5**. (C) The effect of QA structure on the mean open time of the AChR. Data are plotted as mean + SD (n = 3 for each compound).(TIF)

Table S1 **Comparisons of PQ1–5 and some previously reported open-channel blockers for the muscle-type AChR.** The microscopic binding and dissociation kinetics (*k_+B_* and *k_–B_*), affinity (K_d_ = *k_–B_*/*k_+B_*), and molecular complexity (described as the Bertz index) are compared for AChR blockers of diverse structures.
(PDF)

File S1 **A combined file including detailed methods of synthesizing PQ1–5 (including the characterizations of each compound), patch-clamp recording, and analysis of single-channel currents.**
(PDF)
