# Supplementary figures and images for: Poly(Ethylene Glycol) as a Scaffold for High-Affinity Open-Channel Blockers of the Mouse Nicotinic Acetylcholine Receptor
Source: PLoS One. 2014 Nov 11;9(11):e112088. doi: 10.1371/journal.pone.0112088 (PMC4227698; doi:10.1371/journal.pone.0112088)

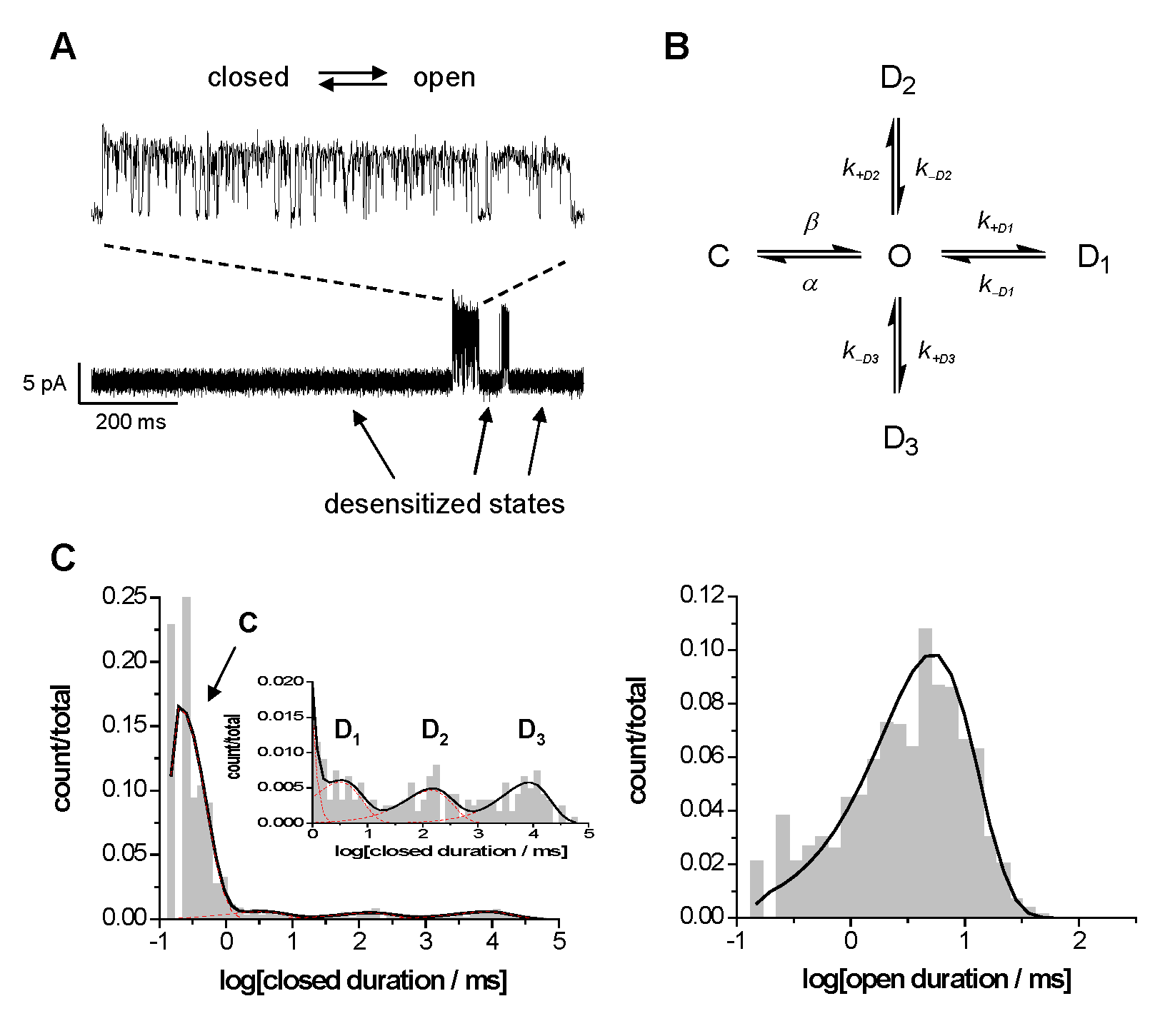

Supplement: Figure S1 — Characterization of single-channel currents at 100 µM ACh (i.e. the control condition). (A) A representative trace showing clusters of opening/closing events separated by desensitized dwells. Currents are shown as upward deflections. Pipette potential was held at +70 mV. (B) The kinetic model used for fitting the acquired single-channel events (by MIL). C, closed state; O, open state; D1, D2, D3, desensitized states (ranked in the order of increasing lifetime). (C) Representative histograms (duration in ms) for the closed-time (left) and the open-time (right) distributions. Each component in the closed-time histogram is designated by the corresponding state in the kinetic model. The solid curves represent the overall fit for the histogram, and the red dashed curves indicate the fits for individual components. Additional details about data acquisition and analysis can be found in File S1 (section 1.3). (TIF) [file pone.0112088.s001.tif]

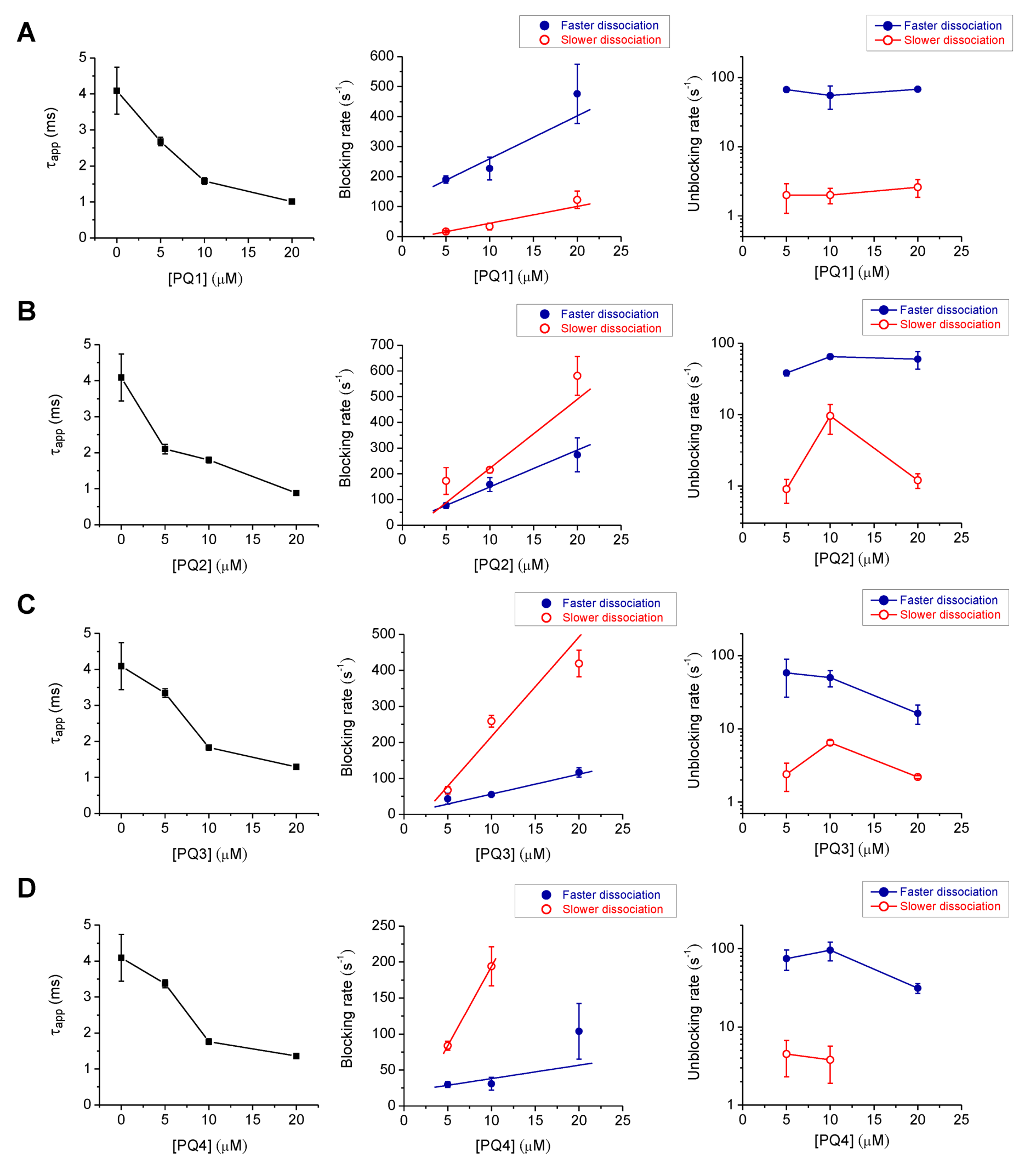

Supplement: Figure S3 — Kinetic characterizations of AChR blockade mediated by PQ1 (A), PQ2 (B), PQ3 (C), and PQ4 (D). Recordings were carried out in the cell-attached configuration held at +70 mV, with 100 µM ACh and various doses of PQ compounds in the recording pipette. The analyses of single-channel currents for PQ1–4 were the same as those for PQ5 (Figure 2 and related text). Each PQ compound caused a dose-dependent decrease in the apparent mean open time (τapp, left) and dose-dependent increases in the relative fractions (but not the duration lifetimes) of two closed-time components. These closed-time components may thus be interpreted as two modes of open-channel blockade. MIL analysis on dwell-time histograms provided the blocking rates (middle) and unblocking rates (right) for these two modes (blue: faster dissociation, i.e. shorter blockade durations; red: slower dissociation, i.e. longer blockade durations). Data are plotted as mean ± SEM (n = 2–4). The blocking rate constants (k+Bs) were obtained from the slopes of least-squares linear fitting for the blocking rates, and the unblocking rate constants (k–Bs) were calculated by averaging the unblocking rates at all tested concentrations. The resulting rate constants are summarized in Figures 2E and 2F. Due to the complexity in closed-time histograms, the data of slower dissociation mode at 20 µM PQ4 were not included in this analysis. (TIF) [file pone.0112088.s003.tif]

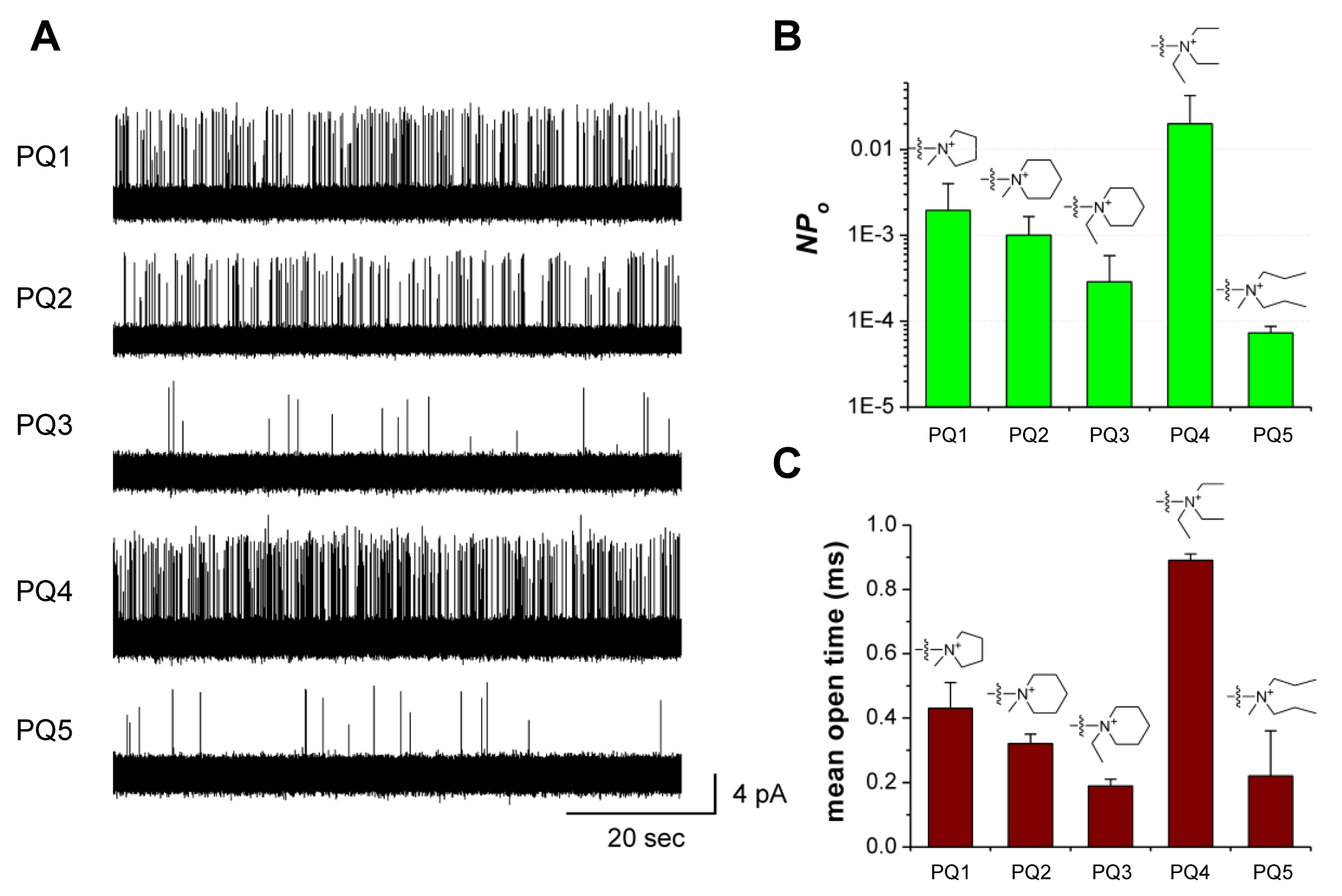

Supplement: Figure S4 — The agonist potencies of PQ1–5. (A) Representative traces of single-channel recordings in the presence of individual PEG-QA (10 µM in the pipette solution without other agonists). Pipette potential was held at +70 mV, and the currents are shown as upward deflections. (B) The effect of QA structure on the open probability of the AChR (defined as , where N max is the maximal number of simultaneously open channels and Po,i is the fraction of time in which i channels open simultaneously). Data are plotted in log-scale in order to present the low NPo values of PQ3 and PQ5. (C) The effect of QA structure on the mean open time of the AChR. Data are plotted as mean + SD (n = 3 for each compound). (TIF) [file pone.0112088.s004.tif]
