# Supplementary material for: Poly(Ethylene Glycol) as a Scaffold for High-Affinity Open-Channel Blockers of the Mouse Nicotinic Acetylcholine Receptor
Source: PLoS One. 2014 Nov 11;9(11):e112088. doi: 10.1371/journal.pone.0112088 (PMC4227698; doi:10.1371/journal.pone.0112088)
Supplement: Table S1 — Comparisons of PQ1–5 and some previously reported open-channel blockers for the muscle-type AChR. The microscopic binding and dissociation kinetics (k+B and k–B), affinity (Kd = k–B/k+B), and molecular complexity (described as the Bertz index) are compared for AChR blockers of diverse structures. (PDF) [file pone.0112088.s005.pdf]

Table S1. Comparisons of PQ1–5 and some previously reported open-channel blockers for the muscle-type AChR

**compound** [a]	k_{+B} ($\mu M^{-1}s^{-1}$)	k_{-B} (s^{-1})	K_d (μM)	**Bertz complexity metric** [c]	**Ref.**
**PQ1**	20 ± 4 [b]	64 ± 14 [b]	3 ± 1	444	--
**PQ2**	29 ± 7 [b]	2 ± 2 [b]	0.08 ± 0.09	471	--
**PQ3**	22 ± 6 [b]	4 ± 2 [b]	0.17 ± 0.12	497	--
**PQ4**	22 [b]	4 ± 3 [b]	0.19 ± 0.15	407	--
**PQ5**	15 ± 1	2 ± 1	0.12 ± 0.09	446	--
(+)-tubocurarine	3	0.8	0.27	1734	1
physostigmine	20	450	23	553	4
lamotrigine	6.2	575	93	522	3
MK-801	20	48	2.4	495	2
QX-314	19	~ 30	~ 1	396	6
ketamine	3	100	33	395	5
QX-222	21	~600	~30	363	6
albuterol	43	1042	24	361	8
procaine	2	200	100	328	10
IEM-1460	72	858	12	306	7
pyrantel	80	667	8	290	9
hexylPIP	22	980	45	204	11
acetylcholine	37	56000	1200	115	12
tetraethylammonium (TEA)	--	--	2700	47	13
choline	--	--	12500	46	14

[a] Compound structures are depicted below.
[b] The values (mean ± SD) for the predominant blockade mode are listed.
[c] The Bertz molecular complexity metric is calculated based on the methods described in refs. 15 and 16.

Structures associated with Table S1:

**PQ1**	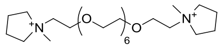
**PQ2**	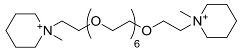
**PQ3**	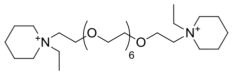
**PQ4**	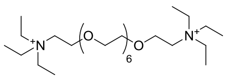
**PQ5**	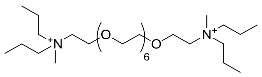
(+)-tubocurarine	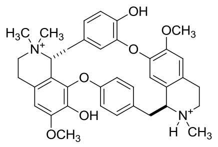
physostigmine	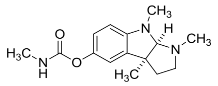
lamotrigine	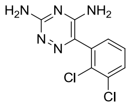
MK-801	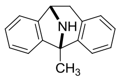
QX-314	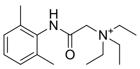
ketamine	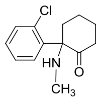
QX-222	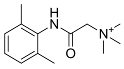
albuterol	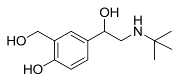
procaine	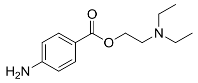
IEM-1460	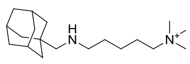

pyrantel	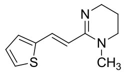
hexylPIP	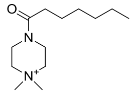
acetylcholine	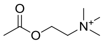
tetraethylammonium (TEA)	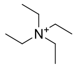
choline	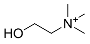

REFERENCES

(1) Bufler J, Wilhelm R, Parnas H, Franke C, Dudel J (1996) Open channel and competitive block of the embryonic form of the nicotinic receptor of mouse myotubes by (+)-tubocurarine. J Physiol 495: 83–95.

(2) Amador M, Dani JA (1991) MK-801 inhibition of nicotinic acetylcholine receptor channels. Synapse 7: 207–215.

(3) Vallés AS, Garbus I, Barrantes FJ (2007) Lamotrigine is an open-channel blocker of the nicotinic acetylcholine receptor. Neuroreport 18: 45–50.

(4) Militante J, Ma BW, Akk G, Steinbach JH (2008) Activation and block of the adult muscle-type nicotinic receptor by physostigmine: single-channel studies. Mol Pharmacol 74: 764–776.

(5) Scheller M, Bufler J, Hertle I, Schneck HJ, Franke C, et al. (1996) Ketamine blocks currents through mammalian nicotinic acetylcholine receptor channels by interaction with both the open and the closed state. Anesth Analg 83: 830–836.

(6) Neher E, Steinbach JH (1978) Local anaesthetics transiently block currents through single acetylcholine-receptor channels. J Physiol 277: 153–176.

(7) Antonov SM, Johnson JW, Lukomskaya NY, Potapyeva NN, Gmiro VE, et al. (1995) Novel adamantane derivatives act as blockers of open ligand-gated channels and as anticonvulsants. Mol Pharmacol 47: 558–567.

(8) Milone M, Engel AG (1996) Block of the endplate acetylcholine receptor channel by the sympathomimetic agents ephedrine, pseudoephedrine, and albuterol. Brain Res 740: 346–352.

(9) Rayes D, De Rosa MJ, Spitzmaul G, Bouzat C (2001) The anthelmintic pyrantel acts as a low efficacious agonist and an open-channel blocker of mammalian acetylcholine receptors. Neuropharmacology 41: 238–245.

(10) Bufler J, Franke C, Parnas H, Dudel J (1996) Open channel block by physostigmine and procaine in embryonic-like nicotinic receptors of mouse muscle. Eur J Neurosci 8: 677–687.

(11) Carter AA, Oswald RE (1993) Channel blocking properties of a series of nicotinic cholinergic agonists. Biophys J 65: 840–851.
(12) Ogden DC, Colquhoun D (1985) Ion channel block by acetylcholine, carbachol and suberyldicholine at the frog neuromuscular junction. Proc R Soc Lond B 225: 329–355.
(13) Akk G, Steinbach JH (2003) Activation and block of mouse muscle-type nicotinic receptors by tetraethylammonium. J Physiol 551: 155–168.
(14) Purohit Y, Grosman C (2006) Block of muscle nicotinic receptors by choline suggests that the activation and desensitization gates act as distinct molecular entities. J Gen Physiol 127: 703–717.
(15) Bertz SH (1981) The first general index of molecular complexity. J Am Chem Soc 103: 3599–3601.
(16) Hendrickson JB, Huang P, Toczko AG (1987) Molecular complexity: a simplified formula adapted to individual atoms. J Chem Inf Comput Sci 27: 63–67.
