# Supplementary material for: Poly(Ethylene Glycol) as a Scaffold for High-Affinity Open-Channel Blockers of the Mouse Nicotinic Acetylcholine Receptor
Source: PLoS One. 2014 Nov 11;9(11):e112088. doi: 10.1371/journal.pone.0112088 (PMC4227698; doi:10.1371/journal.pone.0112088)
Supplement: File S1 — A combined file including detailed methods of synthesizing PQ1–5 (including the characterizations of each compound), patch-clamp recording, and analysis of single-channel currents. (PDF) [file pone.0112088.s006.pdf]

Supporting Information for:

Poly(ethylene glycol) as a Scaffold for High-Affinity Open-Channel Blockers of the Mouse Nicotinic Acetylcholine Receptor

Wan-Chen Lin[1] and Stuart Licht[2*]

Department of Chemistry, Massachusetts Institute of Technology, Cambridge, Massachusetts 02139, USA

[1] Current address: Department of Molecular and Cell Biology, University of California, Berkeley, CA 94720, USA
[2] Current address: Oncology Division, Sanofi US, Cambridge, MA 02139, USA
* Correspondence should be addressed to S.L. (stuart.licht@sanofi.com)

Table of Contents

1. Materials and Methods

1.1. Synthesis of blockers **PQ1**–**5** (Scheme S1)
1.2. Recording of single-channel events
1.3. Analysis of single-channel data (see also: Figure S1)

2. References

1. Materials and Methods

1.1. Synthesis of blockers PQ1–5

Scheme S1. Synthesis of PEG-QA conjugates **PQ1**–**PQ5**.

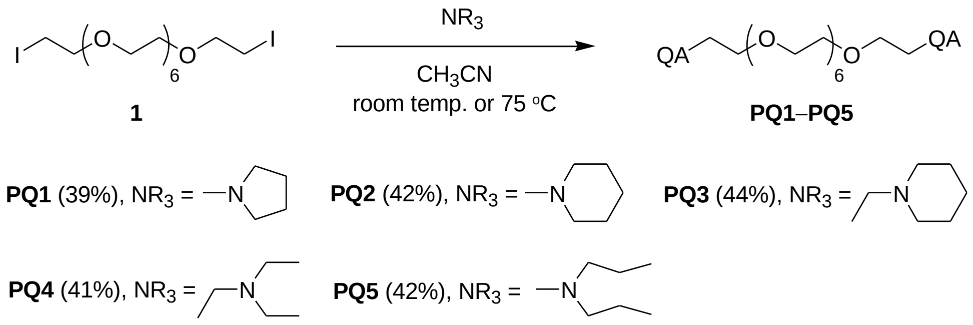

General Methods. Chemicals were purchased from Aldrich (Milwaukee, WI) as reagent grade and were used as supplied. Anhydrous methylene chloride and acetonitrile were packed in Sure/Seal™ bottles and were transferred under nitrogen with a syringe. ^{1}H and ^{13}C NMR spectra were recorded on a Varian Mercury 300 spectrometer. Chemical shifts (δ) were calibrated to the residual solvent peak and were expressed in parts per million (ppm). Coupling constants (*J*) were reported in Hz. High resolution electrospray mass spectra were obtained by a Bruker Daltonics APEXIV 4.7 Tesla FT-ICR mass spectrometer. Analytical thin-layer chromatography (TLC) was performed using silica 60 F_{254}-precoated glass plates (EMD Chemicals Inc., Gibbstown, NJ). Compounds were visualized by staining with aqueous potassium permanganate. Normal-phase column chromatography was carried out on Merck® silica gel 60 (70–230 mesh, Aldrich).

The general synthetic protocol of **PQ1**–**5** is described in the main text (Materials and Methods). Diiodide **1** was prepared following the procedures described in ref. 1.

PQ1: The bis(*N*-methylpyrrolidinium) salt **PQ1** was prepared from diiodide **1** (124 mg, 0.21 mmol) and *N*-methylpyrrolidine. The reaction was carried out at 25 ˚C for 24 h and the crude product was purified by silica gel chromatography (MeOH:$CHCl_3$, 1:2.5 v/v) to provide **PQ1** as a pale yellow solid (62 mg, 39%). ^{1}H NMR (300 MHz, CD_3OD): δ 3.97 (m, 4H), 3.72–3.58 (m, 36H), 3.17 (s, 6H), 2.24 (br m, 8H); ^{13}C NMR (75 MHz, DMF-d_7): δ 71.3 (br, PEG backbone), 71.2, 71.0, 66.0, 65.9, 63.8, 49.4, 22.3; HRMS–ESI (*m/z*): $[M – I]^+$ calcd. for $C_{26}H_{54}IN_2O_7$, 633.2970; found: 633.2977.

PQ2: The bis(*N*-methylpiperidinium) salt **PQ2** was prepared from diiodide **1** (139 mg, 0.24 mmol) and *N*-methylpiperidine. The reaction was carried out at 75 ˚C for 16 h and

the crude product was purified by silica gel chromatography (MeOH:$CHCl_3$, 1:3 v/v) to provide **PQ2** as a pale yellow solid (79 mg, 42%). ^{1}H NMR (300 MHz, CD_3OD): δ 3.98 (br m, 4H), 3.74–3.62 (m, 28H), 3.55 (m, 4H), 3.50 (m, 4H), 3.22 (s, 6H), 1.94 (br m, 8H), 1.72 (m, 4H); ^{13}C NMR (75 MHz, DMF-d_7): δ 71.3 (br, PEG backbone), 71.2, 70.9, 65.1, 63.0, 62.5, 49.8, 21.8, 20.8; HRMS–ESI (*m/z*): $[M - I]^+$ calcd. for $C_{28}H_{58}IN_2O_7$, 661.3283; found: 661.3256.

PQ3: The bis(*N*-ethylpiperidinium) salt **PQ3** was prepared from diiodide **1** (124 mg, 0.21 mmol) and *N*-ethylpiperidine. The reaction was carried out at 75 ˚C for 18 h and the crude product was purified by silica gel chromatography (MeOH:$CHCl_3$, 1:2.5 v/v) to provide **PQ3** as a pale yellow solid (76 mg, 44%). ^{1}H NMR (300 MHz, CD_3OD): δ 3.92 (br m, 4H), 3.70–3.54 (m, 32H), 3.54 (m, 4H), 1.92 (m, 8H), 1.72 (m, 4H), 1.33 (t, *J* = 7.2 Hz, 6H); ^{13}C NMR (75 MHz, DMF-d_7): δ 71.3 (br, PEG backbone), 71.0 (2 peaks), 65.0, 60.2, 58.0, 56.0, 22.0, 20.6, 8.1; HRMS–ESI (*m/z*): $[M – I]^+$ calcd. for $C_{30}H_{62}IN_2O_7$, 689.3596; found: 689.3611.

PQ4: The bis(triethylammonium) salt **PQ4** was prepared from diiodide **1** (141 mg, 0.24 mmol) and triethylamine. The reaction was carried out at 75 ˚C for 24 h and the crude product was purified by silica gel chromatography (MeOH:$CHCl_3$, 1:2.5 v/v) to provide **PQ4** as a pale yellow solid (77 mg, 41%). ^{1}H NMR (300 MHz, CD_3OD): δ 3.89 (br m, 4H), 3.70–3.62 (m, 24H), 3.51 (m, 4H), 3.44 (q, *J* = 7.2 Hz, 12H), 1.31 (t, *J* = 7.2 Hz, 18H); ^{13}C NMR (75 MHz, CD_3OD): δ 71.7, 71.6 (br, PEG backbone), 71.5, 65.6, 57.9, 55.0, 8.2; HRMS–ESI (*m/z*): $[M – I]^+$ calcd. for $C_{28}H_{62}IN_2O_7$, 665.3596; found: 665.3583.

PQ5: The bis(*N*-methyldipropylammonium) salt **PQ5** was prepared from diiodide **1** (124 mg, 0.21 mmol) and *N*-methyldipropylamine. The reaction was carried out at 75 ˚C for 17 h and the crude product was purified by silica gel chromatography (MeOH:$CHCl_3$, 1:2.5 v/v) to provide **PQ5** as a pale yellow solid (72 mg, 42%). ^{1}H NMR (300 MHz, CD_3OD): δ 3.92 (br m, 4H), 3.72–3.62 (m, 24H), 3.59 (m, 4H), 3.36 (m, 8H), 3.13 (s, 6H), 1.80 (m, 8H), 1.02 (t, *J* = 7.2 Hz, 12H); ^{13}C NMR (75 MHz, DMF-d_7): δ 71.2 (br, PEG backbone), 71.0, 65.3, 64.6, 61.8, 49.6, 16.7, 11.1; HRMS–ESI (*m/z*) $[M – I]^+$ calcd. for $C_{30}H_{66}IN_2O_7$, 693.3909; found: 693.3915.

1.2. Recording of single-channel events

Single-channel currents from adult mouse muscle AChRs were recorded using patch-clamp techniques in the cell-attached configuration. For dose-dependence measurements, a holding potential of +70 mV was applied. Patch pipettes were pulled from borosilicate capillary tubes (World Precision Instruments, Sarasota, FL) and were coated with Sylgard 184 (Dow Corning, Midland, MI). Pipette tips were typically 0.5–1 μm in diameter. The extracellular solution (bath solution) comprised (mM): 137.9 NaCl, 8.1 Na_2HPO_4, 2.7 KCl, 1.5 KH_2PO_4, 0.9 $CaCl_2$, and 0.5 $MgCl_2$ (pH 7.4). The pipettes were filled with bath solution supplemented with the tested chemical(s). The currents

were amplified (Axopatch 200B, Axon Instruments, Foster City, CA), low-pass filtered at 10 kHz, and digitized at 20 kHz (NI 6040 E Data Acquisition Board, National Instruments, Austin, TX). Data were recorded directly to a desktop PC hard drive using the QuB software (www.qub.buffalo.edu).

1.3. Analysis of single-channel data (see also: Figure S1)

Kinetic analysis of single-channel currents was carried out using the QuB suite [2, 3]. The currents were idealized using the segmented k-means (SKM) hidden Markov algorithm at full bandwidth (10 kHz) [4]. Kinetic modeling of the idealized intervals was performed using the maximum interval likelihood (MIL) method [2, 3] with a dead time of 0.125 ms. The values of apparent mean open time (τ_{app}) were calculated by MIL. Because the blockade kinetics were determined in the presence of 100 μM ACh, a model (Figure S1B) which includes both channel gating and desensitization is used for analysis (see description below). Recording segments containing more than 500 opening events but no more than two simultaneously open channels were chosen for analysis.

At 100 μM ACh, the AChR is mostly desensitized (i.e. the channel binds agonists with a high affinity but assumes a non-conducting conformation). Once recovered from desensitization, the AChR undergoes cycles of opening and closing (due to rapid re-activation of the closed channel by high dose of ACh) before re-entering the desensitized state. Single-channel currents are displayed as clusters of openings separated by long closed dwells (Figure S1A). The long dwells are desensitization periods, while the brief gaps within the clusters represent channel closing events. The observed closed-time distribution requires at least four exponential components for MIL fitting, which is consistent with the previously reported observations (ref. 5 and references therein). The lifetimes of these components are: <1 ms (fastest component), ~5 ms, ~50 ms, and ~1 s (slowest). In some recordings where channel activity is very low, a fifth component (lifetime >1 s) is present. Here we designate the closed-time components as C (closed, lifetime <1 ms), D_1 (fast desensitized, lifetime ~5 ms), D_2 (intermediate desensitized, lifetime ~50 ms), and D_3 (slow desensitized, lifetime ~1 s) states in our kinetic model for MIL fitting (Figures S1B and S1C). The histogram of the open durations can be reasonably fitted by a single-exponential distribution (Figure S1C).

If an open-channel blocker follows a simple sequential blockade mechanism:

$$C \underset{\alpha}{\overset{\beta}{\rightleftharpoons}} O \underset{k_{-B}}{\overset{k_{+B}[B]}{\rightleftharpoons}} OB \qquad \text{(Eq. 1)}$$

(where C, O, and OB refer to the closed, open, and blocked states of the AChR, and β, α, k_{+B}, k_{-B}, and [B] refer to channel opening rate, channel closing rate, blocking rate constant, unblocking rate constant, and blocker concentration, respectively), a dose-dependent decrease in the apparent mean open time and the presence of a “blocked” component in the closed-time histogram will be observed. The relative area of the blocked component increases with increasing blocker concentration, but the lifetime (τ_B)

of this component is independent of blocker concentration. As shown in Figure S1C, the events in the closed-time histogram arise mainly from the brief channel-closing dwells (C) at 100 μM ACh and the relative areas of the desensitized components are small. If τ_B is larger than the lifetime of the closed-state component (C), the blocked component can be easily identified. Moreover, fitting the closed- and open-time histograms (by MIL) provides the transition rates between states. The blocking rate (i.e. the rate at which the open channel enters the blocked state) is predicted to be proportional to the blocker concentration, but the unblocking rate is concentration-independent. The dose-dependent effects of each PEG-QA on the AChR currents were studied through the analysis of the open- and closed-time distributions as well as the microscopic rates estimated from MIL fitting. The results of single-channel analysis for **PQ1**–**5** are summarized in Figure 2 (main text), Figure S3, and Table S1.

2. References

(1) Lin WC, Licht S (2008) Polymer-based open-channel blockers for the acetylcholine receptor: the effect of spacer length on blockade kinetics. Biochemistry 47: 9163–9173.

(2) Qin F, Auerbach A, Sachs F (1996) Estimating single-channel kinetic parameters from idealized patch-clamp data containing missed events. Biophys J 70: 264–280.

(3) Qin F, Auerbach A, Sachs F (1997) Maximum likelihood estimation of aggregated Markov processes. Proc R Soc Lond B 264: 375–383.

(4) Qin F (2004) Restoration of single-channel currents using the segmental k-means method based on hidden Markov modeling. Biophys J 86: 1488–1501.

(5) Prince RJ, Pennington RA, Sine SM (2002) Mechanism of tacrine block at adult human muscle nicotinic acetylcholine receptors. J Gen Physiol 120: 369–393.
